# Supporting Older Adults’ Understanding of Health Research: Insights from Community Engagement Studios

**DOI:** 10.1093/geroni/igaf122.4359

**Published:** 2025-12-31

**Authors:** Samuel Van Vleet, Nicholas Cloney, Ruochen Yang, Alice Xie, Kristina Brackpool, Hugo Aparicio, Evan Plys, Jin hui Joo

**Affiliations:** Massachusetts General Hospital | Harvard Medical School, Boston, Massachusetts, United States; Brigham & Women’s Hospital, Boston, Massachusetts, United States; Massachusetts General Hospital, Boston, Massachusetts, United States; University of Nevada, Las Vegas, Las Vegas, Nevada, United States; Mass General Brigham, Santa Clarita, California, United States; Boston University, Boston, Massachusetts, United States; Massachusetts General Hospital, Boston, Massachusetts, United States; Massachusetts General Hospital, Boston, Massachusetts, United States

## Abstract

Dementia research studies often struggle to recruit and retain participants from ethnic minority groups. Community Engagement (CE) studios have been used to facilitate discussion within a population of interest to obtain their perspectives. Presentations by experts on relevant topics also draw listeners, so combining the two may increase participation in the studios. To conduct a public engagement program using CE studios to transfer health and research knowledge and to understand the community’s views on research and research participation, eleven modified CE studios were conducted in older adult housing authorities. These studios included presentations on relevant health-related topics to encourage turnout. In addition, study staff interviewed eight resource coordinators and followed up several months later to confirm initial analyses and gather additional suggestions. Through the CE studies, information on health topics such as dementia, loneliness and mental health were provided. Attendees of the CES and the resource coordinators shared their perceptions of health research, described barriers that currently limit research participation and provided a number of recommendations for improving community partnerships. By implementing these modified CE studios in communities of low-income older adults, researchers could obtain new insights on how research is viewed and what can be done to increase participation within these groups. Modified Community Engagement Studios may be a valuable tool for improving participation compared to traditional CE Studios.

